# Advances in immunotyping of colorectal cancer

**DOI:** 10.3389/fimmu.2023.1259461

**Published:** 2023-10-09

**Authors:** Yinhang Wu, Jing Zhuang, Zhanbo Qu, Xi Yang, Shuwen Han

**Affiliations:** ^1^ Huzhou Central Hospital, Affiliated Central Hospital HuZhou University, Huzhou, China; ^2^ Key Laboratory of Multiomics Research and Clinical Transformation of Digestive Cancer of Huzhou, Huzhou, China; ^3^ Huzhou Central Hospital, Fifth Affiliated Clinical Medical College of Zhejiang Chinese Medical University, Huzhou, China

**Keywords:** colorectal cancer, immunotherapy, immune subtype, tumor immune microenvironment, colorectal cancer subtypes

## Abstract

Immunotherapy has transformed treatment for various types of malignancy. However, the benefit of immunotherapy is limited to a minority of patients with mismatch-repair-deficient (dMMR) and microsatellite instability-high (MSI-H) (dMMR-MSI-H) colorectal cancer (CRC). Understanding the complexity and heterogeneity of the tumor immune microenvironment (TIME) and identifying immune-related CRC subtypes will improve antitumor immunotherapy. Here, we review the current status of immunotherapy and typing schemes for CRC. Immune subtypes have been identified based on TIME and prognostic gene signatures that can both partially explain clinical responses to immune checkpoint inhibitors and the prognosis of patients with CRC. Identifying immune subtypes will improve understanding of complex CRC tumor heterogeneity and refine current immunotherapeutic strategies.

## Introduction

Colorectal cancer (CRC) is a prevalent global malignancy with an increasing incidence and high mortality that affected 1.93 million patients and resulted in 0.94 million deaths during 2020. Predictions indicate that China and the USA will have the most new CRC diagnoses over the next 20 years (1). Although the incidence and mortality of CRC can be significantly reduced by screening, metastases occur in 15%-30% of diagnosed CRC patients, while 20%-50% develop metastases during the course of the disease (2). Patients with metastasis CRC (mCRC) have a poor prognosis, with a median overall survival (OS) of only 25–30 months (3, 4). Coupled with the fact that the benefits of chemotherapy and targeted therapy are limited, effective treatment strategies for treating CRC remain difficult to develop.

Immunotherapy has been remarkably successful in eliminating malignant cells by harnessing the immune system to fight cancer (5, 6). Current immunotherapies for cancer include tumor vaccines, monoclonal antibody therapy, cytokine therapy, adoptive immunotherapy, oncolytic virus therapy, and immune checkpoint inhibitors. Soumya Badrinath et al. developed a novel tumor vaccine targeting MICA and MICB stress proteins expressed due to DNA damage, which effectively enhanced the immune system’s ability to recognize and kill cancer cells by enhancing the density of MICA/B proteins on the surface of cancer cells (7). Additionally, the successful combination of tumor vaccines and bacteria through synthetic biology technology, such as the application of flagellate bacteria or bacterial vesicles to deliver tumor antigens, has moved toward the process of targeting tumor sites, prompting the production of *in-situ* tumor vaccines (8, 9). Monoclonal antibody is the most widely used in clinical practice. Recent studies have optimized and improved the variable region of antibody to improve the efficacy and specificity of monoclonal antibody (10, 11). Cytokines such as interferon, interleukin and tumor necrosis factor are involved in the regulation of tumor microenvironment and anti-tumor immune response. Widely studied cytokines and their receptors are playing a key anti-tumor role in the field of cancer immunotherapy. For example, a novel engineered Interleukin-2 (IL-2) fusion protein called ALKS 4230, performed well in a mouse lung tumor model and improved antitumor efficacy, showing strong pharmacokinetic and selective pharmacodynamic properties (12). Adoptive immunotherapy transfers activated or killing immune cells into cancer patients, thereby enhancing the body’s anti-tumor immunity. At present, adoptive immunotherapy has been effectively combined with CRISPR/Cas9 technology to improve the therapeutic function of immune cells and the ability to recognize antigens (13). As a promising immunotherapy agent, oncolytic viruse can accurately dissolve tumor cells, produce tumor antigens *in situ*, and induce anti-tumor specific immune responses. In a phase 1b clinical trial of 21 melanoma patients, the combination of the immunotherapy drug pembrolizumab and an oncolytic virus called T-VEC achieved a 62 percent response rate, better than when treated alone (14). The results showed that oncolytic virus had a great potential to regulate the tumor microenvironment and positively support the anti-tumor immune response of the body. Immune checkpoint inhibitor (ICI) therapy can rejuvenate T cells and modulate the adaptive immune system to prevent immune escape controlled by multiple checkpoints, such as cytotoxic T lymphocyte (CTL)-associated protein 4 (CTLA-4) and programmed cell death ligand 1 (PD-L1) (15–17). An immune checkpoint blockade (ICB) confers prolonged benefits on some patients with advanced cancer and a considerably improved disease prognosis (18, 19).

Although immunotherapy has remarkably changed the treatment landscape of many malignancies, its benefits in terms of CRC are limited to that which is mismatch-repair-deficient (dMMR) and microsatellite instability-high (MSI-H) (dMMR-MSI-H) (20, 21). This type of CRC is associated with abundant immune cell infiltration and tumor mutation accumulation and accounts for 15% of all CRC. The programmed cell death protein 1 (PD1) antibodies pembrolizumab and nivolumab and the combination of nivolumab and ipilimumab (a cytotoxic T-lymphocyte associated protein 4 (CTLA4) antibody) have elicited effective and durable responses, and their use for treating CRC has been approved by the US Food and Drug Administration (FDA) (22). However, immunotherapy currently offers little or no clinical benefit to 85% of patients with mismatch-repair proficient (pMMR) and microsatellite instability-low (MSI-L) or microsatellite stable (MSS) (pMMR-MSI-L/MSS) CRC (20). The prognosis is worse for patients with pMMR-MSI-L/MSS, than dMMR-MSI-H CRC (23). Therefore, the main challenge is to refine immunotherapeutic strategies to treat pMMR-MSI-L/MSS CRC. Another important challenge is pseudoprogression during immunotherapy that manifests as a transient increase in the tumor burden followed by delayed tumor shrinkage that is not true tumor progression (24). With the widespread application of immunotherapy for cancer, the accurate discrimination of pseudoprogression from true progression is critical to help clinicians avoid prematurely stopping immunotherapy and initiating alternative strategies.

The prognosis of patients with CRC is variable even among those with the same tumor stage, and the tumor heterogeneity of CRC significantly impacts the effects of immunotherapy. The tumor microenvironment (TME) comprising tumor, stromal, and immune cells, as well as cytokines, chemokines, and extracellular matrix (ECM), plays essential roles in tumor initiation, progression, immunity and immunotherapy (25–27). Immune cells within the TME of CRC have better predictive value for survival than the tumor-node-metastasis (TNM) classification system (28) and MSI status (29). Therefore, in-depth exploration of the immune landscapes in CRC might help to understand the complex tumor heterogeneity, which will lead to the design of more appropriate therapies and the development of novel immunotherapeutic strategies.

Recent focus on immune-related classification has provided more accurate CRC subtype data that have helped to tailor clinical treatment strategies. In 2015, four consensus molecular subtypes (CMSs) was reported by the international CRC Subtyping Consortium, which was identified based on six independent classification systems and each CMS had distinct molecular and immune features (30). Consensus molecular subtype 1 (CMS1) is MSI immune and accounts for 14% of all CMSs. This subtype is enriched in ~ 76% of MSI tumors and is characterized by frequent B-Raf proto-oncogene serine/threonine protein kinase (P94; BRAF) mutations and powerful immune activation. The canonical subtype, CMS2, accounts for 37% of all CMSs. The WNT and MYC signaling pathways are profoundly activated in this subtype. Epithelial tumors are characterized by significant metabolic dysregulation and frequent KRAS mutations in metabolic CMS3, which accounts for 13% of all CMSs. Tumors in mesenchymal CMS4 have high epithelial-mesenchymal transition (EMT) ability, stromal infiltration, angiogenesis, and account for 23% of all CMSs. Nonetheless, these molecular subtypes were determined based on disease biology rather than clinical outcomes, and their predictive value awaits further investigation.

Clinically important immune subtypes of CRC that respond to immunotherapy have recently been explored to develop more precise therapeutic strategies (**Table 1**). For instance, four immune subtypes have been proposed based on the density of CD3+ and CD8+ T-lymphocytes at the tumor center and margins (36). These cold, altered excluded, altered immunosuppressive, and hot tumor subtypes are characterized by low CD3+/CD8+ at the tumor center and margin, high CD3+/CD8+ at the tumor margin but little at the center, low CD3+/CD8+ at the tumor center or margin, and high CD3+ and low CD8+ at both the tumor center and margin. T-cell exclusion and dysfunction play crucial roles in preventing cytotoxic T lymphocytes (CTLs) from killing tumor cells in the tumor immune microenvironment (TIME) (37). T-cell activity evaluated as CD3+ and CD8+ T-lymphocyte density at the tumor center and margins, is a prognostic biomarker in patients with early-stage CRC, which is independent of TNM stage and MSI status for predicting prognosis (38, 39). The four immune subtypes were associated with a distinct relapse risk of CRC (36). CRC has been clustered into high, medium and low immune infiltration subtypes based on the immune landscape of the TME (40). These subtypes have different immune infiltration levels, programmed cell death 1 ligand 1 (PD-L1) expression, and survival, providing a basis for CRC stratification and personalized immunotherapy. However, which immunophenotypes are more meaningful for clinical treatment and prognosis has not reached consensus.

**Table 1:** Representing immunophenotyping of CRC.

	Immunological subtype			
**Tang et al.** (31)	high immune dysfunction	low immune activation	potent immune exclusion	intense immune activation and slight immune dysfunction and exclusion
**Yang et al**. (32)	immune-active	immune-desert	stroma-rich	
**Zheng et al.** (33)	immune-activated	immune-suppressed	non-immune	
**Chong et al.** (34)	immune-inflamed	immune-excluded	immune-desert	
**Luo et al.** (35)	ferroptosis-associated subtype 1	ferroptosis-associated subtype 2	ferroptosis-associated subtype 3	(consistent with the immune-desert, -inflamed, and -excluded subtypes)

Here, we review progress in immune subtyping CRC. A thorough investigation of the immune subtypes of CRC should facilitate the selection of patients with CRC for immunotherapy and the development of optimal immunotherapeutic strategies.

### Immune subtyping CRC

#### Development and mechanism of tumor immune subtyping

Immuno-oncology has transformed cancer treatment. An initial attempt was made during the late 19th century to harness the immune system to treat cancer (41). Immunotherapy, especially immune checkpoint inhibitors (ICIs) such as anti-PD-L1 (atezolizumab), and/or anti-PD1 (nivolumab) monoclonal antibodies (mAbs) have remarkably improved the survival of melanoma and lung cancer. However, most patients with mCRC do not derive any benefit from immunotherapy. For example, patients with pMMR-MSI-L tumors do not respond to pembrolizumab (23). This finding concurred with disappointing outcomes of immunotherapy in unselected patients. Furthermore, the CheckMate 142 study found limited responses in pMMR-MSI-L tumors, as only one in 20 patients responded to a combination of PD1 and CTLA4 antibodies (42). Why the same ICIs have different therapeutic effects on tumors prompted exploration of the potential mechanisms involved.

**Figure 1:**
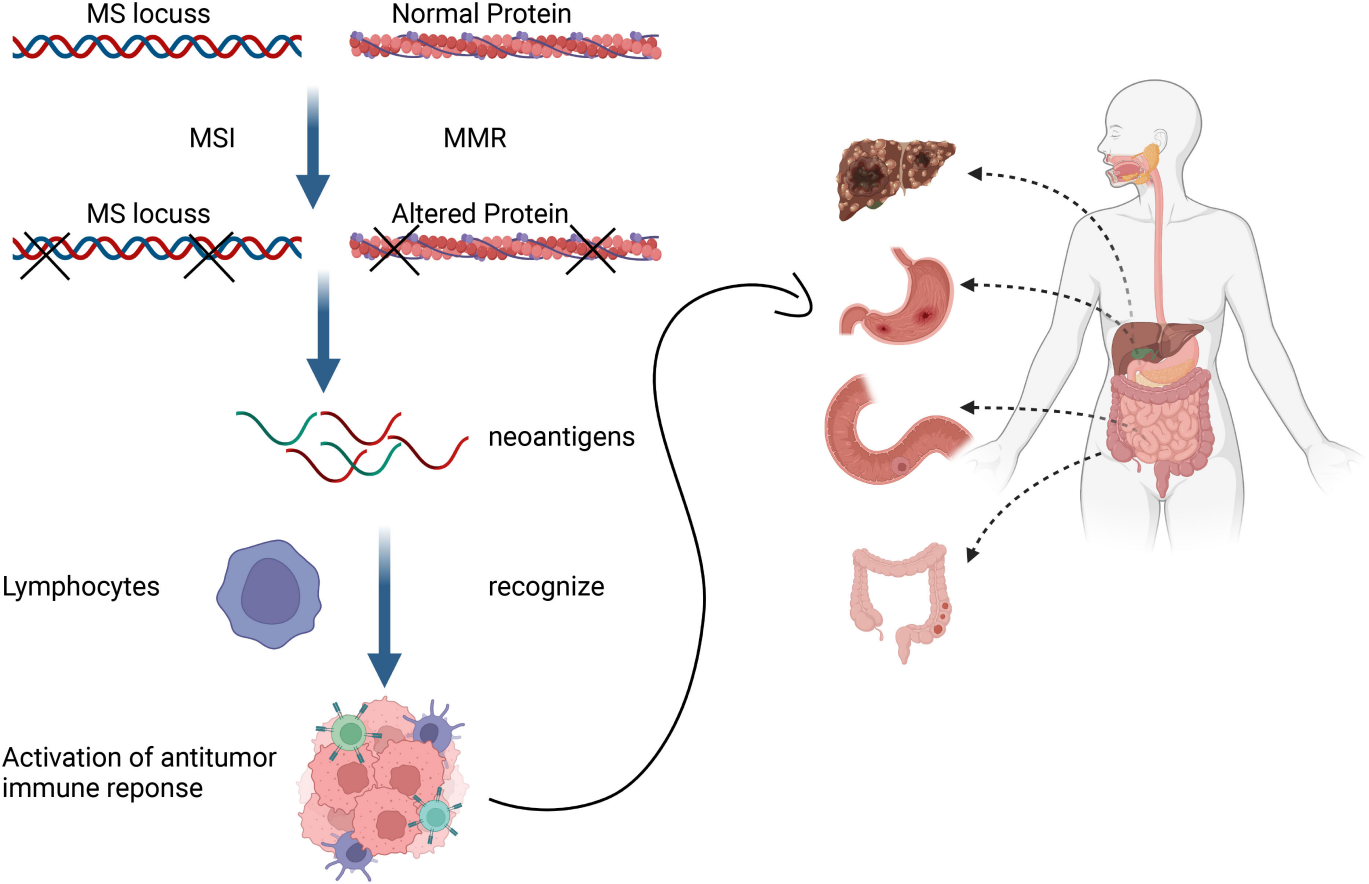
Mismatch repair deficiency triggers anticancer immune responses of different solid tumors by promoting formation of neoantigens that tumor-infiltrating lymphocytes can detect.

The issue was not addressed until the TIME was introduced. The TIME contains abundant T lymphocytes, Th1 helper cells and cytokines such as interferons (IFNs) (43) that facilitate tumor evasion of immune surveillance and thus affect responses to immunotherapy (44). Antitumor responses mediated by the ICI mainly depend on PD-L1 expression in tumors and the infiltration of T cells that can recognize and kill tumor cells (45). Tumors that respond to ICIs generally have higher levels of immune infiltration and/or IFN signatures, and a T-cell-inflammatory phenotype. Tumors such as melanoma with high response rates to ICIs are considered “hot” whereas those with low immune infiltrates such as prostate cancer, are considered “cold” (46). These concepts are the prototypes of tumor immune subtyping. However, tumor classification based on immunophenotypes cannot completely account for the absence of a clinical response to ICIs in many patients. Furthermore, responsiveness to mAbs that block PD-1 is significantly associated with the status of MSI and an MMR deficiency that induce frameshift mutations in tumors. These can lead to the increased formation of neoantigens on tumor cell surfaces (47), which is specifically recognized by tumor infiltrating lymphocytes (TILs) and this triggers anticancer immune responses in different solid tumors (**Figure 1**) (48).

**Figure 2:**
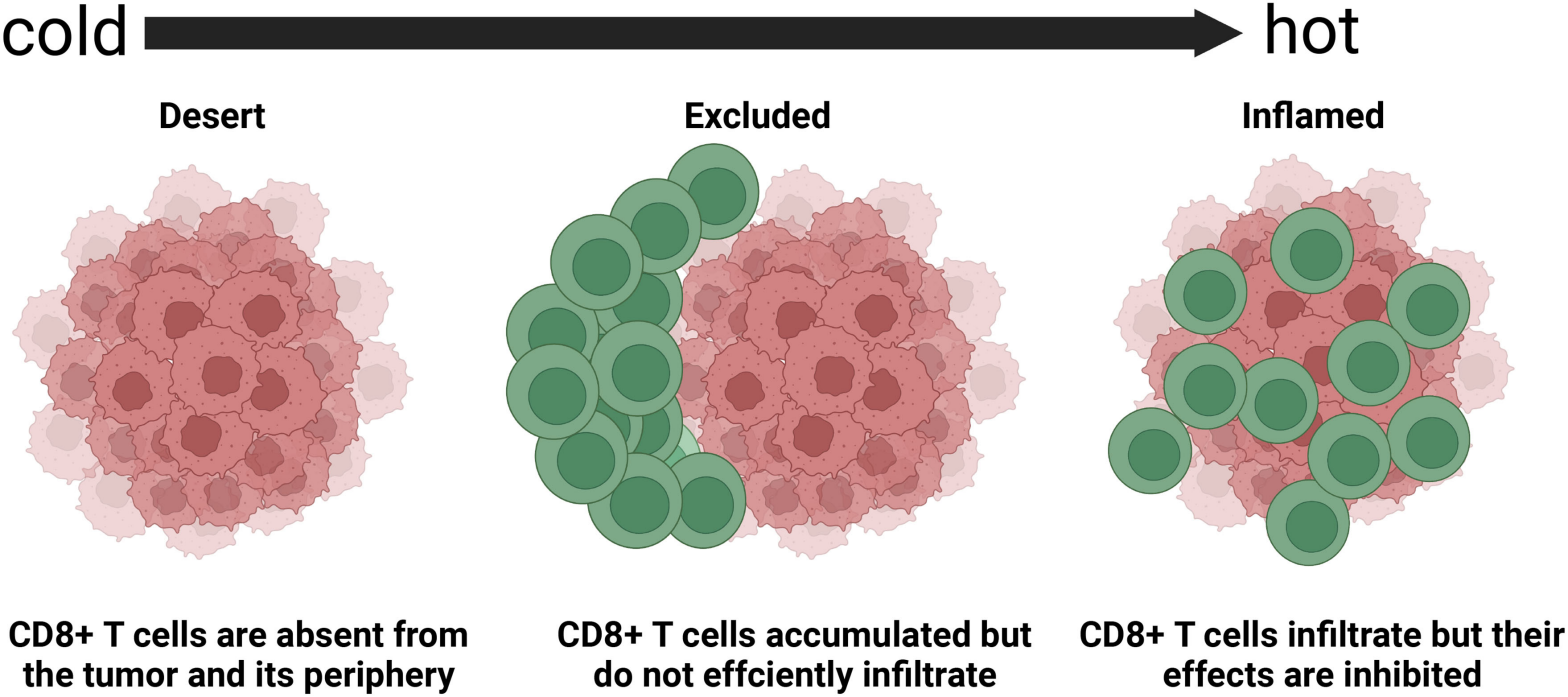
Basic immune-desert,–excluded, and -inflamed phenotypes of tumors determined based on spatial distribution of cytotoxic immune cells in tumor microenvironment (45). Response rates to immune checkpoint inhibitors differ among these phenotypes.

**Figure 3:**
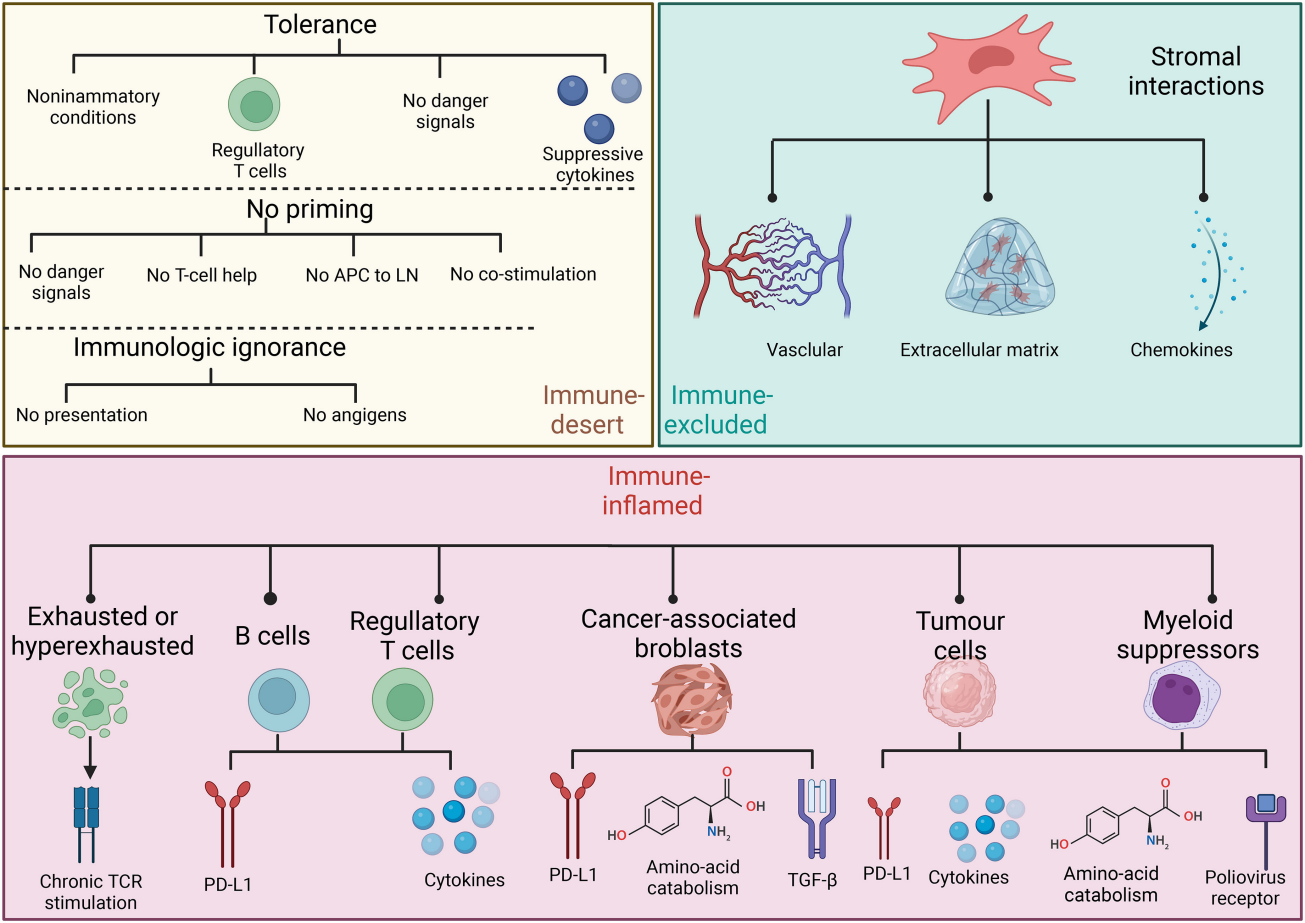
Specific biological mechanisms of three basic immune phenotypes that might prevent host immune response from eliminating cancer.

Considering the heterogeneity of the TIME, many patients experience little or no clinical benefits (objective response) from the same immunotherapy. The complexity of the TIME and the immune subtypes of tumors that are critical for implementing appropriate corresponding immunotherapy have been investigated in detail. Immune-inflamed, -desert, and -excluded tumor immunophenotypes (**Figure 2**) have been proposed based on infiltration by cytotoxic immune cells that are the major components of the TIME (49). **Figure 3** shows the specific biological mechanisms of each of these phenotypes that might prevent the host immune response from eradicating cancer. Immune-inflamed hot tumors have high T-cell infiltration and PD-L1 expression, as well as a larger tumor mutation burden (TMB) (50) that alter the therapeutic effects of ICIs (51–53). Immune-inflamed tumors tend to respond more strongly to ICIs (54, 55). Tumors with immune-desert and -excluded cold phenotypes either lack CD8+ T lymphocytes or cannot efficiently infiltrate tumors (56). Moreover, levels of PD-L1, mutational load, and major histocompatibility complex (MHC) class I expression are low in cold tumors, but are enriched in immunosuppressive tumor-associated macrophages (TAMs) and T-regulatory cells (Tregs) (50). These features are responsible for rare responses of immune-desert and -excluded tumors to ICIs (25). The three proposed tumor phenotypes can help to predict the responses to and effects of immunotherapy on cancer. However, many tumors are already advanced by the time of initial diagnosis and immunohistochemical staining to classify all immunophenotype-based tumors in all patients is unfeasible. Immune classifications from different perspectives of TIME have been focused to guide future targeted immunotherapies. Similar TIME classifications have been proposed according to tumor-infiltrating lymphocyte content and PD-L1 expression (57, 58). Tumors have been categorized as adaptive immune resistant (TIL+/PD-L1+; like hot tumors), immune ignorant (TIL-/PD-L1; like cold tumors), intrinsic inductive (TIL-/PD-L1+; like altered tumors), and immune tolerant (TIL+/PD-L1-; like altered tumors). These could predict responses to immunotherapy and consequential outcomes. Although these immune classifications spanned traditional cancer classifications in terms of anatomical site of origin, the proportions of immune cell infiltration and the prognostic impact substantially varied among immune subtypes. More methods are needed to identify more immune subtypes to better predict and stratify patients who are likely to benefit from immunotherapy.

#### Analysis of immune subtypes based on TIME signatures

The TIME plays essential roles in tumor initiation and responses to therapy. Predicting responsiveness to an ICB based on TIME-related gene expression data from low-resolution sources has improved the efficiency of current ICBs and the design of next-generation immunotherapies (27). Subtypes based on gene expression have been developed as a novel approach to disease stratification and predicting therapeutic responses (59, 60). Therefore, several studies (**Table 2**) have been devoted to immune subtype recognition and corresponding immune escape mechanisms to better predict the benefits of treating CRC.

**Table 2:** Characteristics of studies in this meta-analysis.

Study	n	Subtype name	Datasets
Tang et al. (31)	4	Immune-dysfunctional; immune-inactive; immune-excluded immune-amenable	TCGA, GSE39582
Yang et al. (32)	3	Immune-active, immune-desert, and stroma-rich	GSE39582, GSE14333, GSE33113, GSE17538, GSE39084, GSE38832, GSE37892, KFSYSCC; TCGA-COAD and TCGA-READ
Zheng et al. (33)	3	Immune-activated, immune-suppressed, and non-immune	TCGA-COAD, GSE39582, GSE14333, GSE17538
Chong et al. (34)	3	m^6^A-C1, m^6^A-C2, and m^6^A-C3 (immune-inflamed, immune-excluded, and immune-desert)	TCGA-COAD, GSE39582, GSE14333, and GSE37892
Luo et al. (35)	3	FAC1, FAC2 and FAC3 (immune-inflamed, immune-excluded, and immune-desert)	GSE39582, GSE14333, GSE37892, TCGA-COREAD, and GSE144735

Tang et al. (31) analyzed datasets from the Cancer Genome Atlas (TCGA) (618 patients with CRC) and GSE39582 microarray (316 patients with CRC) (61) from the Gene Expression Omnibus (GEO). The characteristics of TIME were then estimated using the Tumor Immune Dysfunction and Exclusion (TIDE) algorithm. Thereafter, CRC was classified using unsupervised clustering based on PD-L1 expression and CTLs, myeloid-derived suppressor cells, cancer-associated fibroblasts, and M2-like tumor-associated macrophages. The respective characteristics of subtypes 1, 2, 3, and 4 are high immune dysfunction, low immune activation, potent immune exclusion, and intense immune activation and slight immune dysfunction and exclusion. Subtype 1 has potent T-cell infiltration but expresses the most immune checkpoints that might suppress CTL function and was thus considered immune-dysfunctional. Subtype 2 has low CTL infiltration and immune-checkpoint expression and is therefore referred to as immune-inactive. Subtype 3 features high levels of exclusion-markers, indicating potent T-cell exclusion, and is defined as immune-excluded. Subtype 4 has high CTL infiltration, moderate immune checkpoint and exclusion marker expression, and is regarded as immune-amenable. Moreover, the prognosis of subtype 4 is the best among the four subtypes, in line with predictions based on immune subtype characteristics. These immune subtypes could predict different responses to treatments and prognoses, thus facilitating refined personalized immunotherapy for patients with different immune subtypes.

Yang et al. (62) named three TME cell (TMEC) subtypes of CRC based on TME cell infiltration, as immune-active, immune-desert, and stroma-rich subtypes. That study analyzed 1,802 samples from the GSE39582 (61), GSE14333 (63), GSE33113 (64), GSE17538 (65), GSE39084 (66), GSE38832 (67), GSE37892 (68), and KFSYSCC datasets and 619 from the TCGA-COAD and TCGA-READ datasets. The infiltrative abundance of 31 types of cells in the TME was analyzed using the ssGSEA algorithm. The TMEC subtypes were identified using an unsupervised clustering -nonnegative matrix factorization (NMF) algorithm. The immune-active subtype had highly activated adaptive immune cell infiltration, low stromal cell infiltration, and contained more CMS1 and dMMR/MSI-H subtypes. Theoretically, this subtype was believed to benefit the most from ICIs due to an increased abundance of immunosuppressive cells, high levels of immunosuppressive cytokines, and elevated expression of immune inhibitors. The immune-desert subtype contained more CMS2 subtypes and low infiltration of most TME cells. The stroma-rich subtype had more CMS4 subtypes and high immune and stromal cell infiltration. Excessive stromal cell infiltration in this subtype might exclude activated adaptive immune cells from a tumor, resulting in poor survival.

Zheng et al. (33) analyzed data from 488 patients with CRC from the TCGA-COAD dataset (training cohort) and from 1,015 in the GSE39582 (61), GSE14333 (63), and GSE17538 (65) datasets (validation cohorts). They identified immune-related immune-activated, immune-suppressed, and non-immune CRC subtypes using the NMF algorithm based on the top 150 exemplar genes in immune profiles. Stromal-related signatures and immunosuppressive cells were less enriched, prognoses were good, and responses to anti-PD-1 immunotherapy were positive in the immune-activated subtype. Stroma related signatures, immunosuppressive cells, genes, and signaling were activated in the immune-suppressed subtype.

Chong et al. (34) analyzed TCGA-COAD and GSE39582 (61), GSE14333 (63), and GSE37892 (68) microarray datasets. The expression of 23 m6A regulators was analyzed and distinct m6A modifications were identified by consensus clustering with NMF. Three m6A-related subtypes were identified and their TME characteristics were quite consistent with the immune-inflamed, -excluded, and -desert phenotypes. Moreover, a scoring scheme that can quantify m6A modifications in individual tumors could predict clinical responses of patients with CRC to ICIs.

Luo et al. (35) integrated GSE39582 (61), GSE14333 (63), and GSE37892 (68) microarray datasets with TCGA-COREAD, and single-cell RNA (scRNA) sequencing of the GSE144735 (69) dataset to analyze interactions between ferroptosis-related subtypes and TME characteristics. Three ferroptosis-associated subtypes (FAC1, FAC2 and FAC3) identified using the unsupervised clustering NMF algorithm, were consistent with the immune-desert, -inflamed, and -excluded subtypes, respectively. Based on ferroptosis phenotype-related signature, Fersig scoring that associated with survival and immune responses was established. These ferroptosis-associated immune phenotypes facilitated the prediction of responses to immunotherapy in patients with CRC and guide clinical practice for patients with different subtypes.

**Figure 4:**
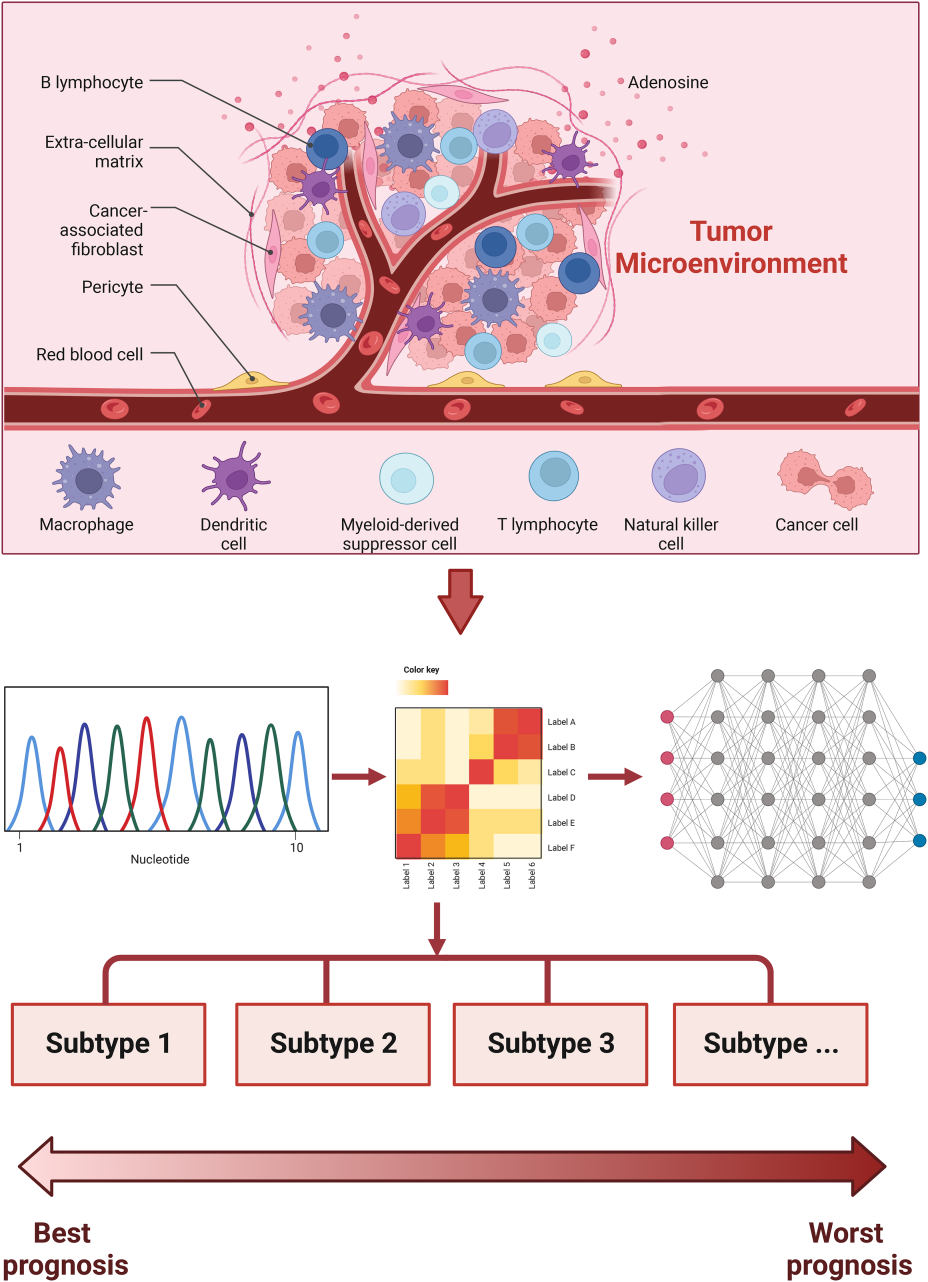
Immune subtype studies based on tumor immune microenvironment signature.

Taken together, these studies screened immune subtypes based on TIME signatures then analyzed their responses to immunotherapies (**Figure 4**). The immune features of different TIME-based immune subtypes provided novel insights into the management of different immune subtypes by tailoring immunotherapy strategies.

#### Analysis of immune subtypes based on prognostic signatures

The prognosis of cancer is associated with tumor immune infiltration (32, 70). Some reliable prognostic biomarkers have been applied to stratify survival risk and predict subtype-specific therapeutic strategies. For example, Wen et al. (71) established a prognostic model using eight immune-related genes, which could predict the 1-, 3-, and 5-year overall survival (OS) of patients with CRC and immun¬e cell infiltration. Xu et al. (72) established an immune-related (IR) lncRNA signature of colon cancer based on eight pairs of immune-related long noncoding (IRlnc) RNA. These had good prognostic value for patients with colon cancer, with an area under the receiver operating characteristic (ROC) curve (AUC) of 0.776 at 1 year.

Patients with CRC have been stratified into different immune subtypes based on the prognostic model. Gene expression and survival information derived from 1,281 CRC samples of independent CRC TCGA cohorts (GSE103479, GSE8832, and GSE87211) were analyzed to establish an immune-related prognostic signature (73). Based on this signature, low- and high-risk was identified in prognostic immune subtypes using the NMF algorithm. Patients with the low-risk subtype were more sensitive to therapy with ICBs, suggesting that subtypes selected based on prognostic immune-related genes could help to guide precise immunotherapy for CRC. An immune-associated miRNA prognostic signature (IAMIPS) comprising immune-related miRNAs (miR-194-3P, miR-216a-5p, and miR-3677-3p) is a powerful independent predictor for the OS of patients with CRC (74). High- and low-risk patients have little and abundant immune cell infiltration and are regarded as having immune-cold and -hot phenotypes, respectively. These had implications for the implementation of immunotherapy in CRC. A prognostic signature based on 16 prognostic immune-related genes stratified patients with CRC into low and high risk groups with higher and lower proportions of immune cell infiltration, respectively, that could guide immunotherapy (75).

**Figure 5:**
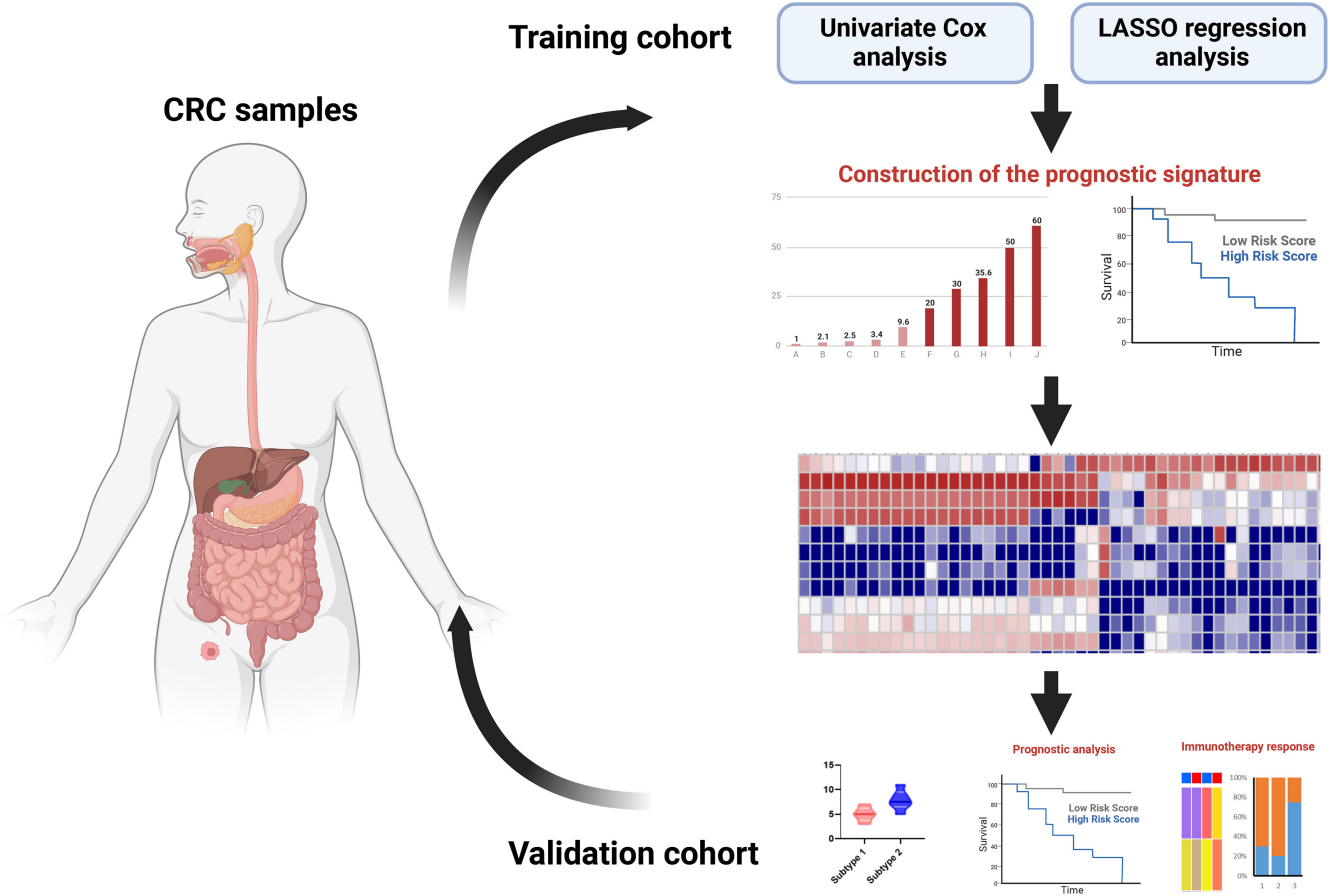
Immune subtype studies based on prognostic signatures.

These studies first analyzed prognostic genes, then constructed prognostic signatures to stratify risk and predict responses to immunotherapy (**Figure 5**). Therefore, prognostic tools might help to optimize immunotherapies for cancer.

#### Prospect of drug selection based on immune subtypes of CRC

Targeted therapy, particularly immunotherapy, has improved outcomes for patients with CRC, especially those with MSI tumors. Immune subtypes of CRC that can predict clinical outcomes of patients with immunogenicity are considered as a promising approach to develop novel drugs for improving immune responses to antitumor agents (38). Immune subtypes determined based on the composition of the TIME have shed new light on patient care and have provided predictive and prognostic factors for immunotherapy such as ICIs (76, 77).

Considering immunogenicity, tumors with different immune subtypes might use different mechanisms to escape immune surveillance (78), suggesting that personalized immunotherapy will improve the outcomes of CRC. The immune-active subtype has abundant immunosuppressive cells, high levels of immunosuppressive cytokines, and elevated expression of immune inhibitors (62). Strategies such as decreasing immunosuppressive cell infiltration and reducing immunosuppressive cytokines might activate tumor immunity, thus improving the immunotherapeutic effects on the immune-active subtype. The immune-desert subtype has low antigen presentation gene expression and low immunogenicity. Therefore, increasing the immunogenicity of this tumor subtype might enhance immune cell chemotaxis, and transform cold, into hot tumors. Due to high immune and stromal cell infiltration in the stroma-rich subtype, TGF-β signaling inhibitors can prevent interactions among cancer cells and the TME avoids the progression of stromal-enriched CRC tumors with a poor prognosis (79). Targeting immunosuppressive cytokines, TGF-β, and/or fibroblasts might transform the stromal-enriched subtype into an immune-active subtype and improve the clinical benefits of immunotherapy. Similarly, the immune features of subtypes described by Tang et al. (31) also provides clues to tailoring treatment strategies. The immune-dysfunctional subtype is likely to benefit from ICBs, whereas responses of the immune-inactive and -excluded subtypes might be elevated by immune agonists and immune-exclusion inhibitors, respectively. Overall, a better understanding of immune subtypes will guide drug selection in future personalized immunotherapy and improve the survival of patients with CRC.

### CRC-related immunotherapy

As mentioned above, CRC presents different heterogeneities and multiple complex subtypes, and each subtype is characterized by different genetic and epigenetic alteration, and is not a trait-unitary disease. Although the majority of CRC patients have resectable lesions, targeted therapy combined with chemotherapy (with oxaliplatin or irinotecan) is the main treatment strategy for patients with advanced CRC. However, the current first-line chemotherapy regimen causes more than just severe side effects, such as gastrointestinal reactions, immune system damage, and even myelosuppression. And the presence of multiple subtypes and heterogeneity also makes CRC response to chemotherapy suboptimal (80). Therefore, adjuvant therapies with fewer side effects and better outcomes are constantly being explored by researchers.

We now know that the immune microenvironment of CRC is becoming the most important tool for understanding the relationship between a patient’s immune system and cancer, prompting researchers to stratify patients by transcriptome-defined subtypes based on cell-type-specific gene expression patterns in cancer (76). The analysis of CRC subtypes is akin to labeling merchandise, and tracing these “labels” has led to the emergence of immunotherapy, an effective treatment with fewer side effects, which is now considered the fifth pillar of treatment after surgery, chemotherapy, radiotherapy and targeted therapy (81, 82).

### Immune checkpoint therapy

Immune checkpoints are a class of immunosuppressive molecules that regulate the strength and breadth of the immune response, thereby avoiding damage and destruction of normal tissues. Immune checkpoints are one of the main causes of immune tolerance in the process of tumor development and progression. Immune checkpoint therapy is a therapeutic method to kill tumor cells by regulating T cell activity through a series of pathways such as co-inhibition or co-stimulation signals. Activated CD4 and CD8 T cells express immune checkpoint receptors such as PD-1 or CTLA-4, which are frequently activated in TME and are responsible for suppressing T cell-mediated immune responses.

The efficacy of using anti-PD-1 immune checkpoint inhibitors in CRC has been validated by several researchers. The activity of the PD-1 inhibitor pembrolizumab was tested in a phase 2 trial that enrolled 11 and 21 CRC patients with dMMR and pMMR, respectively (23). Twenty-week objective response and PFS rates were 40% and 78%, respectively in patients with dMMR tumors, and 0% and 11% respectively in patients with pMMR tumors. The 20-week objective response and PFS rates for patients with dMMR tumors were 40% and 78% respectively, while the 20-week objective response and PFS rates for pMMR tumor patients were 0% and 11% respectively. The researchers identified mutation-associated neoantigens in CRC patients with dMMR tumors that were far superior to those in patients with pMMR tumors. This result was considered not only that dMMR tumors might benefit from anti-PD-1 immune checkpoint inhibitor therapy, but also further supported the notion that mutation-associated neoantigen recognition is a key component of the endogenous anti-tumor immune response.

### Adoptive cell therapy

Adoptive cell therapy (ACT) is an important form of tumor immunotherapy. Autoimmune cells are collected from the human body, cultured *in vitro*, proliferated, or enhanced in their targeted killing function, and then injected into the patient to kill pathogens, cancer cells, and mutated cells in the blood and tissues.

In a phase I clinical trial in CRC patients, genetically engineered T cells were transferred to three patients with metastatic CRC (83). After treatment, all three patients experienced a significant decrease in serum CEA levels (74%-99%) and even regression of tumors that had metastasized to the lungs and liver in one of the patients. However, all three patients developed severe transient inflammatory colitis. Additional clinical studies are needed to demonstrate the true benefit of CAR T cells in human CRC. TIL therapy consists of T cells that target multiple antigens in cancer cells, thereby stimulating a cytotoxic response against the cancer cells through multiple targets (84). Tran reported on a CRC patient treated with TIL targeting the KRAS-G12D mutation (85). Following treatment, six of the seven metastatic foci in the patient’s lungs regressed significantly. As a result, the researchers further screened for CD8+ T cells that specifically recognized mutated KRAS-G12D, further demonstrating the feasibility of TIL therapy.

### Tumor vaccine

Tumor vaccines introduce tumor antigens into patients in various forms (such as tumor cells, tumor-related proteins or peptides, and genes expressing tumor antigens, etc.) to overcome tumor-induced immunosuppression, enhance immunogenicity, activate the patient’s own immune system, and induce cellular and humoral immune responses of the body, so as to achieve the purpose of controlling or eliminating tumors (86).

Several prospective trials have investigated the role of tumor vaccines for CRC. In a recent phase 2 clinical study the efficacy of a tumor vaccine was compared with placebo in patients with mCRC. In this trial, researchers observed a tumor-specific immune response to the vaccine, but the study was terminated early because patients in the vaccine group showed no benefit in median progression-free survival or median overall survival (87). Tumor vaccines, in combination with cancer therapies such as chemotherapy, radiation and ICIs, could potentially be an ideal approach to address tumor immunosuppression.

## Discussion

Heterogeneous CRC has a high therapeutic need. Proficient mismatch repair-microsatellite (pMMR-/MSS tumors) that comprise ~ 95% of mCRC (88), do not meaningfully respond to conventional immunotherapies such as checkpoint blockades, vaccination, and adoptive cell transfer. Tumor cells with low antigenicity or an immunosuppressive TME can counteract antitumor immunity, which might be a key mechanism of immunotherapeutic resistance in most patients with CRC (89). The immune phenotype has more important implications for risk stratification and response predictions than the CMS classification (90). Immune subtyping is required to determine which subtypes would be suitable for ICI treatment, to overcome resistance to immunotherapy, and develop treatment strategies for different CRC subtypes.

A better understanding of the immune landscape of cancer promotes the progress of immune subtyping. The following immune subtypes were defined in 2018: C1 (wound healing), C2 (IFN-γ dominant), C3 (inflammatory), C4 (lymphocyte depleted), C5 (immunologically quiet), and C6 (TGF-β dominant) by analyzing > 10,000 tumors derived from 33 types of cancer (90). Immune subtypes can span anatomical locations and tumor types, but the proportions of the immune subtypes substantially vary among tumor types. CRC comprises mostly C1–C4 subtypes and most CRC is enriched in the C1 subtype that is characterized by a high proliferation rate, elevated angiogenic gene expression, and a Th2 cell bias towards adaptive immune infiltration (91). Novel TIME-based classifications have been investigated to identify more immune subtypes and suitable candidates for innovative immunotherapy. For instance, subtypes based on the TIME of bladder urothelial carcinoma samples in the TCGA database, comprise activated, exhausted, and non-immune groups (92). Patients with the immune-activated subtype had low genetic alterations and were likely to benefit more from anti-PD-1 immunotherapy, whereas those with the immune-exhausted subtype might achieve a good outcome of ICB therapy combined with a TGF-β or EP300 inhibitor. Three immune subtypes of stomach adenocarcinoma have similarly been classified based on distinct TIME signatures and therapeutic responses (93). Among them, subtype 3 was characterized by increased immune T-cell immune cytolytic activity and Th1/IFNγ scores, better immune scores and an improved prognosis compared with the other two subtypes. Immune activated and immunosuppressive subtypes of gastric cancer have also been identified (94). The immune activation subtype had genomic characteristics consistent with responders to anti-PD-1 therapy and had a favorable prognosis. The immunosuppressive subtype was associated with a lack of a response to checkpoint blockade therapy. Thus, anti-PD-L1 combined with anti-TGF-β therapy might be appropriate for this suppressive subtype. The characteristics of immune infiltration have been explored in tumors stratified into different risk groups based on prognostic factors. For instance, three immune subtypes have been determined based on a signature comprising six immune types of cells associated with the prognosis of uveal melanoma (95). Immune subtype 3 had the highest LDA score that reflected a response to anti-PD1 immunotherapy, where immune subtype 1 had the lowest LDA score reflecting an immunosuppressive phenotype. An 11-lncRNA signature that could predict the prognosis of breast cancer was associated with the infiltration of immune cell subtypes (96). Thus, the literature basically shows that immune subtyping has been based on unsupervised cluster analysis of TIME signatures or by analyzing relationships between prognostic signatures and immune characteristics. Immune subtypes obtained from the two perspectives can predict cancer prognosis and immunotherapeutic responses. Immune subtypes determined by analyzing TIME signatures and immune responses of CRC might be more favorable to guide clinical treatment. This is because the prognosis of patients might be determined by many factors, and an immune subtype with a good prognosis is not necessarily sensitive to immunotherapy. Therefore, we believe that immune subtypes based on TIME signatures and immune responses might have greater clinical significance for guiding precise therapy for CRC. Immune subtypes based on TIME signatures are notably similar to the immune-desert, -inflamed, and -excluded subtypes that have value for guiding precise therapy for CRC. However, assigning samples to specific immune subtypes identified using unsupervised clustering is difficult and limits the clinical application of TMEC subtypes. Developing a trained classifier for immune subtyping and validating it in large-scale clinical cohorts might help to improve CRC immunotherapy.

Notably, different immune cells in the TME display distinct sensitivities to ferroptosis, which is an iron-dependent form of cell death that plays significant roles in various diseases including CRC (97–99). Moreover, ferroptosis can modulate antitumor immunity by interacting with various types of immune cells, such as CD8+ T cells (100, 101) that regulate ferroptosis during cancer immunotherapy. Ferroptosis inducers can impact distinct functions on cancer immunity, thus affecting the efficiency of ICIs (102) in tumors with different immunophenotypes (101). This confirmed that the ferroptosis-related immunophenotypes are in line with the three basic immune phenotypes and could predict the prognosis and responses of patients to CRC immunotherapy (35). A better understanding of the characteristics of ferroptosis-associated phenotypes in TME might enhance the effects of current immunotherapies. In addition, modifications play vital roles in mediating innate immunity and antitumor effects by controlling diverse m6A regulators (103). Modifications of m6A interact with TME immune cell infiltration. Based on the expression of 23 m6A regulators, the TME characteristics of three m6A modifications were similar to the basic immune-inflamed, -excluded, and -desert phenotypes, and have high value in predicting the clinical responses of patients with CRC to ICIs (34). These data suggest that the m6A modification could shape TIME profiles and direct therapeutic intervention plans for CRC. Furthermore, the gut microbiota alters the efficacy of cancer immunotherapy, particularly ICIs (104–106). The gut microbiota can promote chemokine production by CRC cells and modulate T cell trafficking into human CRC tumor tissues (107). Identification of immune subtypes based on the gut microbiota might be a novel strategy for improving the effectiveness of cancer immunotherapy.

In conclusion, the tumor heterogeneity of CRC affects responses to immunotherapy. Classifying CRC tumors into different immune subtypes might improve understanding of the complexity of tumor heterogeneity and lead to the development of novel immunotherapeutic strategies. The ability of different immune subtypes to predict immunotherapeutic outcomes requires further exploration.

## Author contributions

YW: Writing – original draft. JZ: Writing – original draft. ZQ: Visualization, Writing – original draft. XY: Writing – original draft. SH: Writing – original draft.
